# Ask the Parent: Developing a Pediatric Feedback Form for Medical Learners

**DOI:** 10.1177/23821205251327375

**Published:** 2025-03-13

**Authors:** Sarah-Marie Durr, Sanjida Newaz, Susan Petryk

**Affiliations:** 1 12371 Department of Medicine, University of Saskatchewan, Saskatoon, Saskatchewan, Canada; 2 Research Department, 7234 Saskatchewan Health Authority, Regina, Saskatchewan, Canada

**Keywords:** medical education, medical student, pediatrics, patient-feedback, patient-centered care, feedback form

## Abstract

**BACKGROUND:**

Patient-centered medicine prioritizes patients’ perspective to actively involve them in their care. Medical education and assessments must reflect this approach. Current patient feedback forms for medical learners are designed for the adult patient and are thus not suited for pediatrics. We aimed to determine if parents/caregivers and simulated patients (SPs) are willing to provide feedback to medical learners and if learners would be receptive to this feedback. We then identified which specific feedback caregivers, SPs, and learners consider most important.

**METHODS:**

REDCap surveys were emailed to caregivers whose child had been seen at Child and Youth Services in Regina, Saskatchewan, from 2020 to June 2023, and to University of Saskatchewan (USask) SPs. Another survey was sent to USask medical students and family medicine and pediatric residents. Surveys asked what specific feedback each group would most prefer to give (caregivers/SPs) or receive (learners) using a Likert scale to rate importance. Descriptive statistics were computed using R software. The highest-ranked options were combined to form a single questionnaire to be given following a clinical encounter.

**RESULTS:**

All three groups agree that medical learners should receive feedback from sources beyond physicians alone (caregivers: 73.6%, SPs: 89.5%, and learners: 88.9%). The five most highly rated areas for feedback were “explains things clearly,” “involves me in the decisions about the medical plans (for my child),” “addresses my concerns and takes them seriously,” “listens and gives their full attention,” and “did or said anything that made me (or my child) uncomfortable.”

**CONCLUSIONS:**

All three groups overwhelmingly agree that caregivers/patients should provide feedback on learners’ clinical skills, confirming the utility of a pediatric feedback form. The most important areas of feedback identified were consolidated into a user-friendly feedback form consisting of five questions with a Likert-scale rating plus a section for free written narrative feedback.

## Introduction

Within the past few decades, the healthcare system has experienced a paradigm shift moving away from paternalistic care toward a patient-centered approach, where patients are active decision-makers who help guide their treatment.^1^ With an increasing focus on the values and perspectives of the patient, it follows that medical education should be altered to reflect this change.

Patient feedback is an important tool that is still being developed and refined in medical curriculums.^2^ It can be used to assess the qualities of medical trainees that lead to effective patient-centered care, especially as some of these traits cannot be accurately evaluated by a physician preceptor. A study by Barr et al^3^ had clinical tutors and patients complete the same student assessment form; they found that patients and tutors agreed in most areas, except in questions of respect and concern, with patients giving lower scores. Patient feedback has also been shown to be an important tool in promoting professional behaviors for medical trainees. One survey found that students believed they would be more likely to change their behavior if they received feedback from a patient, compared to feedback from self-assessment, clinical colleagues, or other students.^4^

The importance of patient feedback in medical education is clear; however, there is a gap in the literature in obtaining patient feedback in pediatric populations. A systematic review by Finch *et al*,^5^ found that patient feedback research has largely focused on patients who are readily capable of communicating feedback, and there is a lack of research with patients with communication disorders or impairments. Similar to patients with communication difficulties, pediatric patients are often solely reliant on a parent, or more generally their primary caregiver, to express their concerns and give feedback on their care. Although a patient feedback questionnaire for medical students has been developed, it does not directly address the unique concerns of a pediatric patient population.^6^ The parents (parents from here on will be referred to as caregivers) of pediatric patients speak on behalf of their child; they must gauge the feelings of their child and advocate for them. The caregiver is also in direct contact with the medical student or physician and has their own concerns around the care of their child. As such, the medical students or physicians need to both address the concerns of the caregivers, the health concerns of the child, and gain the trust of both parties. For these reasons, a patient feedback form intended for adult patient feedback does not directly translate into pediatrics.

A questionnaire for pediatric inpatients was developed by the Child Hospital Consumer Assessment of Healthcare Providers and Systems, with the intent of quantifying the experience of pediatric patients in hospitals and improving the quality of their care.^7^ However, this questionnaire is not intended to be used for the feedback of medical trainees and is also time-consuming for caregivers to complete, with 62 questions.^7^

During the first two years of medical school, students may encounter more simulated patients (SPs) than real patients. SPs are critical to developing the patient-centered skills necessary for clinical practice, as well as practicing physical examination techniques before entering a real clinical environment. Feedback from SPs can be the first patient-centered feedback medical students receive, and yet there is very little standardization for how this feedback is given.^8^ There are many noted roadblocks for SPs in providing feedback to medical students. Interviews with SPs have shown they do not always know how honest to be with students, especially if they need to give negative feedback.^9^ SPs also found it difficult to provide feedback to students if their opinions differed from that of the clinical preceptor instructing the students.^9^

The existing research on patient feedback is centered on adult patients, and to date, there is no validated tool for collecting pediatric patient feedback for medical trainees. There is an opportunity to develop a tool that can be used in medical curriculums to help students and residents improve their patient-centered skills in pediatrics, a patient population with unique needs compared to adult patients. We intended for our pediatric patient feedback form to be completed by the caregiver of pediatric patients rather than the patients themselves. This is in some ways a practical consideration as infants and young children would not be able to complete a feedback form. However, older pediatric patients who may be capable of communicating how the encounter felt for them, still heavily rely on their caregivers to navigate the healthcare field. Once pediatric patients are able to access healthcare independently, such as teenagers approaching adulthood, they may be more suited to use the previously described patient feedback forms that were trialed on adult populations.

The purpose of this research was to identify the aspects that caregivers and SPs prioritize when providing feedback to medical trainees. Additionally, the research sought to uncover the types of feedback that medical trainees find most beneficial from patients and caregivers. This data would allow us to develop a feedback form that could be used by caregivers or pediatric patients to help supplement the feedback medical trainees receive.

## Methods

### Study design

This study aimed to investigate the perceptions and experiences of caregivers and SPs in providing feedback to medical learners, in order to create a pediatric feedback form. We employed a cross-sectional design. The survey questionnaire included close-ended questions, covering various aspects of an encounter between a medical learner and a pediatric patient (ie, rapport with the child, medial expertise, and communication skills). The reporting of this study conforms to the DoCTRINE (Defined Criteria To Report INnovations in Education) guideline^10^ (Supplemental File 1). The complete survey questionnaire can be found in Supplemental File 2. Ethical considerations were prioritized, and consent was implied. This project received ethics approval (REB-23-17) and operational approval (OA-SHA-23-17) by the Saskatchewan Health Authority Research Ethics Board.

### Settings and participants

Following ethics approval, the caregiver survey was distributed electronically, and data collection took place in June 2023. Caregivers received an email invitation to complete the survey if they had a child seen at the Child and Youth Clinic in Regina, Saskatchewan, from 2020 to June 2023. The medical learners survey was emailed to University of Saskatchewan (USask) medical students, as well as pediatric and family medicine residents. The SP survey was emailed to current SPs who have worked for the College of Medicine, both the Regina and Saskatoon campuses. We included SPs because first- and second-year medical students mostly interact with SPs during their clinical sessions.

## Data Collection

### Demographic survey questions

Caregivers and SPs were asked about their age, gender, ethnicity, geographic residence, and highest level of education. Caregivers were asked to report their child's age and gender, and SPs were asked how long they have worked in the SP program, and if they had worked with medical students, residents, and/or other healthcare students. Medical trainees were asked about their program (medical student, family medicine resident, or pediatric resident) and gender.

### Caregiver survey

Caregivers were asked if they have ever had a medical trainee present during a previous appointment for their child, and if so, how many times they recall this happening, and if any physician has specifically asked them to provide feedback for the trainee. They then responded to a series of statements regarding providing feedback to medical learners, using a six-point Likert scale. Caregivers were then shown a list of 13 attributes that they could assess in medical trainees. They were also asked to select the five most significant qualities on which they would like to provide feedback. The attributes were derived from those most reported in the literature of other feedback questionnaires and from clinician and student experience. There was also space for additional free written comments to ensure important attributes were not missed.

### SP survey

Similar to the caregiver survey, SPs were asked if they have ever been asked to provide feedback for the trainee by the physician preceptor, and if so, how often this occurs. They then responded to a series of statements regarding providing feedback to medical learners, using the same Likert scale. They were also presented with the same list of qualities that they could provide feedback on for medical trainees, with one statement regarding medical trainees interacting with the caregiver's child during the appointment removed.

### Medical trainee survey

Medical trainees were asked about their experience with pediatric patients in the presence of caregivers and whether their preceptors regularly sought feedback from patients or caregivers during clinical encounters, along with the frequency of such occurrences. Trainees then responded to a series of statements regarding getting feedback from patients/caregivers, using the same Likert scale. They were also shown the list of 13 qualities patients/caregivers would be invited to provide feedback on regarding their performance, and they were asked to select the top five most important ones.

### Data analysis

A comprehensive survey data analysis was completed, focusing solely on descriptive statistics. By employing various measures of central tendencies, a thorough exploration of the survey responses was conducted, allowing for a nuanced understanding of the participants’ perspectives. The de-identified data was aggregated. Statistical analysis performed using R software. Continuous variables were summarized using medians and interquartile ranges. Categorical variables were summarized in counts and percentages.

## Results

### Participant characteristics

Email invitations to the respective surveys were sent to 937 caregivers, 150 SPs, 295 medical students, and 173 pediatric and family medicine residents. The response rate for caregivers was 9% (87 responded), 89% for SPs (133 responded), 16% for medical students (47 responded), and 9% for residents (16 responded). Medical student and resident data were analyzed together to reflect “medical learners.” Of the medical learners, 30.2% (19 out of 63) were male, 66.7% (42 out of 63) were female, and 3.2% (2 out of 63) preferred not to answer. For SPs, the median amount of time they have worked as an SP was three years, with an interquartile range of 1-7 years. For the SPs, 93.2% (124 out of 133) have worked with medical students, 66.9% (89 out of 133) with residents, 18% (24 out of 133) with “other” healthcare students, and 7.4% (10 out of 133) were unsure. Demographic data on caregiver and SPs can be found in Table 1.

**Table 1.** Caregivers, child, and SP demographic data.

		CAREGIVER	CHILD	SP
AGE (MEAN ± SD)		45.97 ± 9.29	12.74 ± 4.42	56.5 ± 16.70
Gender	Male	7 (8.0%)	40 (46%)	39 (29.3%)
	Female	67 (77%)	27 (31%)	79 (59.4%)
	Other	1 (1.1%)	1 (1.1%)	4 (3.0%)
	Prefer not say/missing/unclear	1 (1.1%)	9 (10.3%)	11 (8.3%)
Ethnic background	Caucasian	42 (48.3%)		88 (66.2%)
	Indigenous	10 (11.5%)		2 (1.5%)
	Black	3 (3.4%)		1 (0.8%)
	Asian	1 (1.1%)		5 (3.8%)
	Middle Eastern	0 (0%)		1 (0.8%)
	Mixed	3 (3.4%)		1 (0.8%)
	Prefer not say/missing/unclear	11 (12.6%)		35 (26.3%)
Residence	City (urban)	56 (64.4%)		115 (86.5%)
	Rural	18 (20.7%)		7 (5.3%)
	Other	3 (3.4%)		1 (0.8%)
	Missing	10 (11.5%)		0 (0%)
Highest level of education	Less than Grade 12	1 (1.1%)		1 (1.1%)
	Grade 12	9 (10.3%)		22 (16.5%)
	GED	1 (1.1%)		1 (0.8%)
	Trades or business certificate	25 (28.7%)		14 (10.4%)
	Bachelor	22 (25.3%)		47 (35.3%)
	Masters	5 (5.7%)		20 (15%)
	PhD	0 (0%)		2 (1.5%)
	Other	13 (14.9%)		17 (12.8%)

### Caregivers and SPs experience with providing feedback to medical learners

Of the 87 caregivers, 89.7% (78) had had appointment(s) involving a medical learner. For each caregiver, the average number of appointments involved a trainee were 3.42 with a standard deviation of 2.78. During their previous appointments involving learners, only 11.5% (10 out of 87) of caregivers were asked to provide feedback on the performance of the trainee.

Out of 133 respondents, 66.2% (88) of SPs have been requested to provide feedback for the trainees they interacted with, whereas 33.8% (45) have not. Among those asked, 21.8% (29 out of 133) mentioned it occurs rarely, 36.1% (48 out of 133) said sometimes, 6.8% (9 out of 133) said often, and 1.5% (2 out of 133) said always. Responses were not provided by 33.8% (45 out of 133) of the respondents.

All medical learners (100%, 63) have had an encounter with a pediatric patient with the child's caregiver present. During encounters with real patients (ie, not SPs), 52.4% (33 out of 63) learners reported having seen a preceptor ask the patient for feedback on their performance, while 47.6% (30 out of 63) have not. Of those that have had preceptors ask patients for feedback, 22.1% (14 out of 63) said this is rarely done, 25.4% (16 out of 63) said it occurs sometimes, 3.2% (2 out of 63) said often, and 1.6% (1 out of 63) said always.

### Caregivers and SPs keen to provide medical learners feedback

When caregivers were questioned about the prospect of patients and caregivers providing feedback on the performance of medical trainees to enhance their skills, 82.8% (72 out of 87) expressed agreement ranging from slightly agree (9.2%, 8 out of 87), agree (32.2%, 28 out of 87), and strongly agree (41.1%, 36 out of 87). Moreover, 77% (67 out of 87) of caregivers indicated their willingness to complete an anonymous two-min questionnaire after their appointment to evaluate the trainee's performance.

Concerning in-person feedback, 49.4% (43 out of 87) of caregivers agreed that they may not be fully honest when giving direct, in-person feedback as to avoid hurting the trainee's feelings (slightly agree: 19.5%, agree: 18.4%, and strongly agree: 11.5%). Conversely, 80.4% (70 out of 87) of caregivers asserted that they would provide full and honest feedback, even if it were negative, if they were assured of the anonymity of their responses, ranging from 10.3% slightly agreeing, 26.4% agreeing, and 43.7% strongly agreeing. A complete list of statements and caregiver responses can be found in Supplemental File 3.

Of the SPs, 97.0% (129 out of 133) expressed agreement (slightly: 18.0%, agree: 30.8%, and strongly: 48.1%) that they should be giving medical learners feedback. Additionally, 87.2% (116 out of 133) have had sessions where they wanted to give the learner feedback but were not asked by the preceptor. The majority of SPs (92.5%, 123 out of 133) would be willing to fill out a two-min questionnaire on a medical trainee's performance right after the session. 61.7% (82 out of 133) are comfortable disagreeing with the feedback that the preceptor gave a medical trainee (slightly agree: 24.1%, agree: 27.8%, and strongly agree: 9.8%). Similarly, 65.4% (87 out of 133) SPs disagreed that they would not want to give their honest feedback to the medical trainee if it differed from what their preceptor said, ranging from 16.5% slightly disagreeing, 35.5% disagreeing, and 13.5% strongly disagreeing. A complete list of statements and SP responses can be found in Supplemental File 3.

### Medical learners seek patients and/or caregivers feedback

A large majority of medical learners (95.2%, 60 out of 63) slightly to strongly agreed that receiving patient feedback would allow them to improve their patient-centered clinical skills (slightly agree: 9.5%, agreed: 46.0%, and strongly agree: 39.7%), and the same proportion would want to see feedback if a patient was to provide it (slightly agree: 3.2%, agree: 39.7%, and strongly agree: 52.4%). A vast majority of learners (96.8%, 61 out of 63) agreed that patient feedback is valuable and unique from the feedback they usually received from preceptors ranging from 15.9% slightly agreeing, 34.9% agreeing, and 46.0% strongly agreeing, while 98.4% (62 out of 63) agreed patient feedback could make them better doctors (slightly agree: 9.5%, agree: 36.5%, and strongly agree: 52.4%). Lastly, 88.9% (56 out of 63) of learners disagreed that only preceptors and not patients, could provide feedback on patient-centered skills, ranging from 9.5% slightly disagreeing, 49.2% disagreeing, and 30.2% strongly disagreeing. A majority of learners (95.2%, 60 out of 63) also agreed that a numerical score alone is not very helpful and that they would also want some narrative context to the score. 85.7% (54 out of 63) medical learners believe patients would be honest when giving feedback on their clinical skills if they were using an anonymous questionnaire after the session. Lastly, 41.3% (26 out of 63) of learners slightly to strongly agreed that they would be more likely to change their behavior or communication skills if they got feedback from a patient, than if the feedback came from a doctor (slightly agree: 25.4%, agree: 9.5%, and strongly agree: 6.3%). A complete list of statements and medical learner responses can be found in Supplemental File 3.

### Feedback areas desired by caregivers, SPs, and medical learners

Of the provided list of 13 statements, the top five chosen feedback areas by caregivers included the following:

1. “Involves me in the decisions about the medical plants for my child” (62.1%, 54 out of 87).
2. “Explains things clearly” (60.9%, 53 out of 87).
3. “Addresses my concerns and takes them seriously” (56.3%, 49 out of 87).
4. “Spends some time talking or playing with my child to get to know them better” (37.9%, 33 out of 87).
5. Two Statements tied: “Is skillful at examining my child” and “Is knowledgeable” (32.3%, 28 out of 87).

The top five chosen feedback areas among SPs were as follows:

1. “Explains things clearly” (78.9%, 105 out of 133).
2. “Addresses my concerns and takes them seriously” (61.7%, 82 out of 133).
3. “Listens and gives me their full attention” (57.9%, 77 out of 133).
4. “Did or said anything that made me uncomfortable” (54.9%, 73 out of 133).
5. Two Statements tied: “Involves me in the decisions about the medical plans” and “Makes me feel at ease” (36.8%, 49 out of 133).

The top five chosen feedback areas among medical learners were as follows:

1. “Explains things clearly” (92.1%, 58 out of 63).
2. “Addresses my concerns and takes them seriously” (71.4%, 45 out of 63).
3. “Involves me in the decisions about the medical plans” (60.3%, 38 out of 63).
4. “Did or said anything that made me uncomfortable” (57.1%, 36 out of 63).
5. “Makes me feel at ease” (54.0%, 34 out of 63).

**Figure 1.**
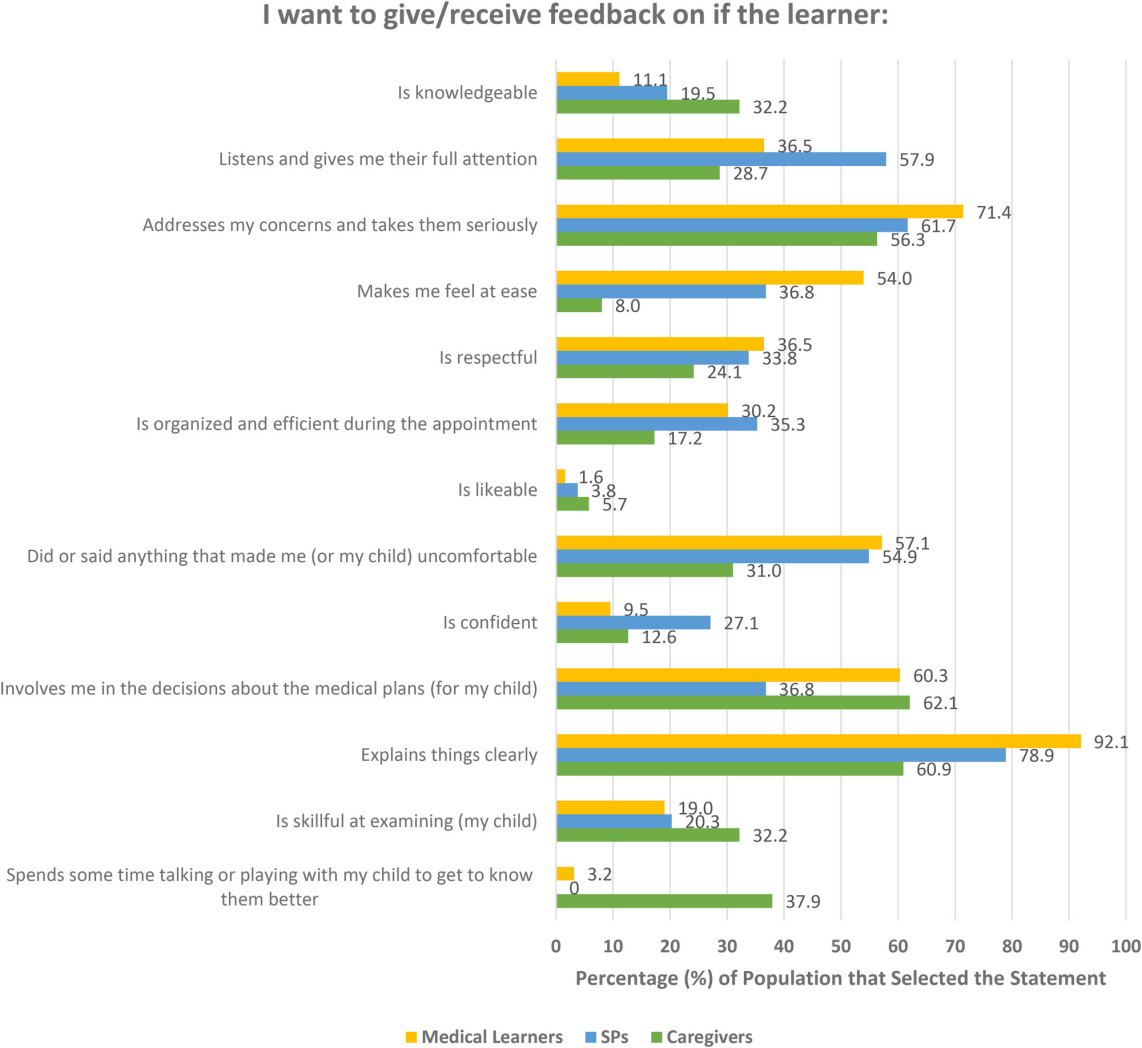
Percentage of times statement was chosen by each population.

A complete list of chosen statements among the three populations can be found in Figure 1.

## Discussion

The majority of caregivers and SPs are willing to provide feedback to medical learners (77%, 67 out of 87 and 92.5%, 123 out of 133, respectively), and in turn, most medical learners (95.2%, 60 out of 63) believe receiving feedback from patients would help them improve their patient-centered skills, which in turn confirms the utility of a pediatric feedback form. The means by which caregivers are asked to provide feedback to medical learners (direct vs anonymous) is important, as it can impact their likelihood of being honest. While informal feedback given in person can be easily elicited, this is infrequently done in appointments with medical learners, with only 11.5% (10 out of 87) of caregivers reporting they have been asked to give feedback, and only 52.4% (33 out of 63) of medical learners have seen a preceptor ask for the patient's input. The frequency of being asked for feedback is higher among SPs, with 66.2% (88 out of 133) reported being asked for their input during clinical sessions; however, only 6.8% (9 out of 133) said this occurs often, and 1.5% (2 out of 133) said it they are always asked.

Previous research has shown that medical students and residents express discomfort if they need to personally ask the patient for feedback.^11–14^ Reasons for discomfort can range from students not wanting to damage the therapeutic relationship they have formed with the patient,^13^ residents who are worried about breaking confidentiality by asking for feedback,^11^ students not wanting the patient to think they were being caring only to get good feedback on their forms for school,^14^ or students not wanting to further burden sick patients.^12^ Many of these concerns could be solved by having a validated questionnaire in place that could be completed anonymously, with the patient/caregiver's consent, that is distributed to the caregivers by a third party (ie, not the medical learner). Our survey also found that caregivers are less likely to provide honest feedback if it is delivered in person, with only 49.4% (43 out of 87) agreeing they would be honest, but if caregivers are given the opportunity to provide feedback anonymously via a feedback form, this percentage increases to 80.4% (70 out of 87). Similarly, 85.7% (54 out of 63) medical learners believed that caregivers would be honest in their feedback if done through a two-min anonymous form.

In terms of what the feedback form should contain, we identified the top five statements selected by each population, and combined the most popular choices, along with those we believed would be most useful for learners. This list includes the following:

1. “Listens and gives me their full attention.”
2. “Addresses my concerns and takes them seriously.”
3. “Explains things clearly.”
4. “Involves me in the decisions about the medical plans for my child.”
5. “Did or said anything that made me or my child uncomfortable.”

Statements #2, #3, and #4 were in the top five statements for all three populations. Statements #1 and #5 were in the top five statements selected by SPs and medical learners. The top statements chosen by medical learners were more heavily favored because the form is intended to provide learners with useful, valuable feedback that they will find helpful to improve their clinical skills. In a similar fashion, because 95.2% (60 out of 63) of medical learners also preferred to have narrative context in addition to a numerical score, we included a free writing section at the end of the feedback form to allow caregivers to explain their thoughts or add further information. A preference for narrative feedback has also been previously demonstrated in literature patient.^6,14–16^ Narrative feedback was considered so critical for high-quality assessments, that previous research found that informally soliciting patient feedback was seen by some students to be more beneficial than a form to complete.^14^

One way our survey results differ from the existing literature is that more than half of the medical learners (57.1%, 36 out of 63) disagreed that they would be more likely to change their communication skills if they received feedback from a patient, than if the feedback came from a doctor (slightly disagree: 33.3%, disagree: 19.0%, strongly disagree: 4.8%). A study of family doctors found they were more likely to make changes in their practice if they received consistent feedback from patients, medical colleagues, and co-workers, or notably, from patients alone.^17^ This difference may be because the family doctors are practicing physicians while we surveyed current medical students and residents who are not yet solely responsible for their patients. In fact, the study of the family doctors explained that they feel most responsible to their patients, so negative feedback from patients was more likely to precipitate change.^17^ However, other research has found that medical students are more likely to change their behavior if they received feedback from a patient, compared to feedback from self-assessment, clinical colleagues, or other students.^4^ The difference with our survey results may be due to the wording of the statement (“I would be MORE likely to change my communication skills if I got feedback from a patient, than if the feedback came from a doctor”) because we asked specifically about feedback coming from a patient or a physician, and not the other sources of feedback mentioned in the study.

Another difference we found was that 61.7% (82 out of 133) of the SPs we surveyed slightly to strongly agreed they would be comfortable disagreeing with the feedback that the preceptor gave a medical trainee (slightly agree: 24.1%, agree: 27.8%, and strongly agree: 9.8%). To confirm this belief, we also asked SPs if they would not want to give their honest feedback to the medical trainee if it differed from what the preceptor said, and 65.4% (87 out of 133) disagreed with this statement, ranging from 16.5% slightly disagreeing, 35.5% disagreeing, and 13.5% strongly disagreeing. In contrast, previous research found SPs said it would be difficult to provide feedback to students if their opinions differed from that of the clinical preceptor who leads the group of students.^9^ This difference may be due to the learning environment, the experience of the SP, and the previous relationship between the clinical preceptors and the SPs. As such, the response as to whether SPs are comfortable disagreeing to the clinical preceptor is highly dependent on the learning context.

A limitation of our study includes the relatively small response rate of caregivers and medical learners (9%, 87 out of 937 and 13.4%, 63 out of 468, respectively), especially compared to the high response rate we had from the SP population (89%, 133 out of 150). While we did receive enough responses for data analysis, it is more difficult to generalize our results to the opinions of all caregivers and medical learners. In addition, the caregivers and guardians that received the invitation for the survey were those whose child has been seen at the Child and Youth Services in Regina, Saskatchewan. This clinic provides mental health services for children up to 11 years old, and performs assessments, treatment, and management for emotional, developmental, and behavioral disorders, among a general pediatric practice. Thus, the sample of caregivers we surveyed may not be representative of all caregivers as their children may have unique mental health or developmental concerns that may not present to a solely general pediatric practice. Nonetheless, this clinic is a learning site for USask medical students and residents, so it was more likely that caregivers would have had an interaction with a medical learner in the past.

Additionally, we are unable to comment on areas such as socioeconomic status of caregivers or SPs, nor if the patients were from a single caregiver household, which are areas that may impact the kind of feedback caregivers/SPs would want to give. These populations may be underrepresented in our data; however, given the nature of the Child and Youth Services clinic and the services provided there, we believe the population would consist of caregivers who have children with complex medical needs.

Our surveys were also only sent out in English; this is the main language that the clinic operates under, as well as the main langue for USask SPs. English is also the most commonly spoken language in Saskatchewan, with it being the first language of 98.1% of the population.^18^ Nonetheless, it may be difficult to use our feedback form in populations other than English speakers, as direct translations of the areas of feedback may not produce the intended meaning.

Involving patients in medical education ensures students are taught patient-centered skills from the beginning of their medical careers, even before they are responsible for their own patients. These skills cannot be underestimated; patient's families have described first-hand how the breakdown of patient-centered care, like transparent communication (encompassed in statement #3 that we identified as an important area of feedback), leads to a breakdown of trust, and thus the therapeutic patient-physician relationship.^19^ Shared decision-making is fundamental to patient-centered care. One intensivist explains how shared decision-making is done by listening to the patient, understanding their goals and perspective, and tailoring their medical plan to align with these values.^20^

Having shown that caregivers are willing to give feedback and that medical learners are willing to receive it, we believe this feedback form designed with the input of caregivers and learners, will be both quick and easy to use for caregivers, and provide learners with valuable, actionable feedback to improve their clinical skills. The next step is to pilot this pediatric patient feedback form in the clinical sessions of USask medical students and residents. This will include using the form during real patient encounters at both general and specialized pediatrician clinics. We will be collecting data and interviewing caregivers on the ease of use of the form, as well as medical learners on the usefulness of the feedback and how this anonymous caregiver-centered feedback compares to the traditional feedback they have received from preceptors. To better understand if the feedback form impacted learners’ approach to pediatric medicine, we will also be asking them about what kind of changes the feedback they received may have had on their history taking or physical exam skills. We hope to further develop the form to create a validated, quick, and easy-to-use feedback form that can be used for any medical learner in any pediatric setting. While the next step in our research process will be to validate the form in USask College of Medicine pediatric clinical encounters, there is potential for the feedback form to be used in other cultural settings beyond Saskatchewan or Canada.

## Conclusion

Caregivers, SPs, and medical learners overwhelmingly agree that caregivers/patients should provide feedback on learners’ clinical skills, confirming the utility of a pediatric feedback form. The most important areas of feedback identified were listening and paying full attention, addressing concerns and taking them seriously, explaining things clearly, involved caregivers in the decision-making, and did or said anything that make the caregiver/child uncomfortable.

## Supplemental Material

sj-docx-1-mde-10.1177_23821205251327375 - Supplemental material for Ask the Parent: Developing a Pediatric Feedback Form for Medical Learners Supplemental material, sj-docx-1-mde-10.1177_23821205251327375 for Ask the Parent: Developing a Pediatric Feedback Form for Medical Learners by Sarah-Marie Durr, Sanjida Newaz and Susan Petryk in Journal of Medical Education and Curricular Development

sj-docx-2-mde-10.1177_23821205251327375 - Supplemental material for Ask the Parent: Developing a Pediatric Feedback Form for Medical Learners Supplemental material, sj-docx-2-mde-10.1177_23821205251327375 for Ask the Parent: Developing a Pediatric Feedback Form for Medical Learners by Sarah-Marie Durr, Sanjida Newaz and Susan Petryk in Journal of Medical Education and Curricular Development

sj-docx-3-mde-10.1177_23821205251327375 - Supplemental material for Ask the Parent: Developing a Pediatric Feedback Form for Medical Learners Supplemental material, sj-docx-3-mde-10.1177_23821205251327375 for Ask the Parent: Developing a Pediatric Feedback Form for Medical Learners by Sarah-Marie Durr, Sanjida Newaz and Susan Petryk in Journal of Medical Education and Curricular Development
