# Supplementary material for: Ask the Parent: Developing a Pediatric Feedback Form for Medical Learners
Source: J Med Educ Curric Dev. 2025 Mar 13;12:23821205251327375. doi: 10.1177/23821205251327375 (PMC11907601; doi:10.1177/23821205251327375)
Supplement: sj-docx-1-mde-10.1177_23821205251327375 - Supplemental material for Ask the Parent: Developing a Pediatric Feedback Form for Medical Learners [file sj-docx-1-mde-10.1177_23821205251327375.docx]

**Defined Criteria To Report INnovations in Education (DoCTRINE)**

*Citation:* Blanco M, Prunuske J, DiCorcia M, Learman LA, Mutcheson B, Huang GC. The DoCTRINE Guidelines: Defined Criteria To Report INnovations in Education. Academic Medicine. 2022 May 1;97(5):689-695.

| **Introduction** | **Y/N** |
| --- | --- |
| 1. Need for the curriculum | Y |
| 1. Review of relevant literature, theories, models, or published curricula | Y |
| 1. Unique contribution of the curriculum to the literature | Y |
| **Curriculum Development** | **Y/N** |
| 1. Purpose/goals of the curriculum | Y |
| 1. Outcome-based learning objectives | Y |
| 1. Target population of learners | Y |
| **Curriculum implementation** | **Y/N** |
| 1. Instructional setting for curriculum delivery | Y |
| 1. Resources for implementing the curriculum | Y |
| 1. Description of instructional methods | Y |
| 1. Methods to evaluate achievement of outcome-based learning objectives | Y |
| 1. Origin of evaluation instrument(s) | Y |
| **Results** | **Y/N** |
| 1. Number of learners participating in the curriculum | Y |
| 1. Number of participants included in the evaluation | Y |
| 1. Evidence of achievement of outcome-based learning objectives | N/A* |
| **Discussion** | **Y/N** |
| 1. Summary of findings | Y |
| 1. Interpretation of findings in relation to the existing literature | Y |
| 1. Lessons learned from the implementation of the curriculum | N/A* |
| 1. Limitations of the evaluation of the curriculum | Y |
| 1. Describes future implications of the curriculum | Y |

*Denotes areas that cannot be evaluated as the form has been developed but not yet implemented in medical education.
