# Supplementary material for: Ask the Parent: Developing a Pediatric Feedback Form for Medical Learners
Source: J Med Educ Curric Dev. 2025 Mar 13;12:23821205251327375. doi: 10.1177/23821205251327375 (PMC11907601; doi:10.1177/23821205251327375)
Supplement: sj-docx-2-mde-10.1177_23821205251327375 - Supplemental material for Ask the Parent: Developing a Pediatric Feedback Form for Medical Learners [file sj-docx-2-mde-10.1177_23821205251327375.docx]

Additional File 1 – Complete Surveys

**Ask the Parent: Developing a Client Feedback Form to Improve Medical Learners’ Pediatric Clinical Skills**

Sarah-Marie Durr^1^, Sanjida Newaz^2^, Susan Petryk^1^

*^1^Department of Medicine, University of Saskatchewan, Saskatoon, Saskatchewan, Canada.*

*^2^Research Department, Saskatchewan Health Authority, Saskatchewan, Canada*

**Parent Survey**

**PART A**

**If you have multiple children at Child and Youth Services, please answer this survey with only one child in mind. If you wish to repeat the survey for any additional children, you may do so by clicking on the link within the original email and taking the survey again.**

**Have you ever had an appointment involving a medical trainee (student or Resident)?**

**Yes**

**No**

If yes, approximately how many times have you seen a medical trainee? _____

**Has anyone ever specifically asked you how the trainee performed or behaved with you in clinic?**

No. I do not recall any times

Yes.

If yes, about how many times do you recall?

Rarely

Sometimes

Often

Always

**How much do you agree with the following statements:**

**Patients and/or their parents should give medical trainees feedback about what they liked and what they could do better to help them become better doctors. Feedback should NOT only come from other doctors or teachers.**

Strongly disagree / disagree / slightly disagree / slightly agree / agree / strongly agree/ N/A

1 2 3 4 5 6

**I think ONLY doctors, should give feedback to medical trainees, and NOT patients or their parents.**

Strongly disagree / disagree / slightly disagree / slightly agree / agree / strongly agree/ N/A

1 2 3 4 5 6

**I have seen some trainees who could really use my feedback to improve their interaction skills.**

Strongly disagree / disagree / slightly disagree / slightly agree / agree / strongly agree/ N/A

1 2 3 4 5 6

**There have been times when I wanted to give feedback to a medical trainee but didn’t because I was not asked.**

Strongly disagree / disagree / slightly disagree / slightly agree / agree / strongly agree/N/A

1 2 3 4 5 6

**I would be willing to fill out a 2-minute anonymous questionnaire on a medical trainee’s performance right after an appointment.**

Strongly disagree / disagree / slightly disagree / slightly agree / agree / strongly agree / N/A

1 2 3 4 5 6

**I might not be fully honest when giving direct, in-person feedback to the medical trainee because I do not want to hurt their feelings.**

Strongly disagree / disagree / slightly disagree / slightly agree / agree / strongly agree / N/A

1 2 3 4 5 6

**I would be completely honest in my feedback, even if it was negative, if I knew it would be anonymous.**

Strongly disagree / disagree / slightly disagree / slightly agree / agree / strongly agree / N/A

1 2 3 4 5 6

**If my child was very sick, I would NOT want to fill out a 2-minute questionnaire on a medical trainee's performance.**

Strongly disagree / disagree / slightly disagree / slightly agree / agree / strongly agree / N/A

1 2 3 4 5 6

**If you gave feedback on a medical trainee, who do you think should see it?**

Only the student / Only the teacher / Both student and teacher

**Are there any other comments you would like to add?**

**PART B**

**Please select what you think are the 5 most important options.**

**If I were giving feedback to a medical student or resident, I would want to comment on if the trainee:**

Spends some time talking or playing with my child to get to know them better

 Is skillful at examining my child

 Explains things clearly

 Involves me in the decisions about the medical plans for my child

 Is confident

 Did or said anything that made me or my child uncomfortable

 Is likeable

 Is organized and efficient during the appointment

 Is respectful

 Makes me feel at ease

 Addresses my concerns and takes them seriously

 Listens and gives me their full attention

 Is knowledgeable

**Which other qualities of a doctor are important to you? _________________**

**We would like to know a bit about you and your child / youth who has been seen at Child and Youth Services.**

Where do you live? _____city (urban) ______rural ______other (please specify)______

What is your age? _____ years

What is your gender? ______male ______female _____other (please specify) ______prefer not to say

What is your highest level of education?

Less than Grade 12

 Grade 12

 GED

 Trades or Business Certificate

 Bachelor

 Masters

 PhD

 Other (please specify) _____________

Which ethnic background do you identify with: ______________________

What is your child’s age? _______ years

What is your child’s gender? ____male ____female _____other (please specify) ______prefer not to say

**SP Survey**

**PART A**

**How long have you been a part of the Simulated Patient program? _____years**

**I have been a Simulated Patient for medical trainees at the following stages (select all that apply):**

**Medical students**

**Resident**

**Other (please specify) _______**

**Not sure**

**Has anyone ever specifically asked you how a trainee performed or behaved with you in your session?**

No. I do not recall any times

Yes.

If yes, about how often do you recall ?

Rarely

Sometimes

Often

Always

**How much do you agree with the following statements:**

**Simulated Patients should give medical trainees feedback about what they liked and what they could do better to help them become better doctors. Feedback should NOT only come from other doctors or teachers.**

Strongly disagree / disagree / slightly disagree / slightly agree / agree / strongly agree/ N/A

1 2 3 4 5 6

**I think ONLY doctors, should give feedback to medical trainees, and NOT Simulated Patients.**

Strongly disagree / disagree / slightly disagree / slightly agree / agree / strongly agree/ N/A

1 2 3 4 5 6

**I have seen some medical trainees who could really use my feedback to improve their interpersonal skills.**

Strongly disagree / disagree / slightly disagree / slightly agree / agree / strongly agree/ N/A

1 2 3 4 5 6

**There have been times when I wanted to give feedback to a medical trainee but didn’t because I was not asked.**

Strongly disagree / disagree / slightly disagree / slightly agree / agree / strongly agree/ N/A

1 2 3 4 5 6

**I would NOT want to give my honest feedback to the medical trainee if my opinion was different from what their preceptor said.**

Strongly disagree / disagree / slightly disagree / slightly agree / agree / strongly agree/ N/A

1 2 3 4 5 6

**I would be willing to fill out a 2-minute questionnaire on a medical trainee’s performance right after a session.**

Strongly disagree / disagree / slightly disagree / slightly agree / agree / strongly agree/ N/A

1 2 3 4 5 6

**I would be comfortable disagreeing with the feedback that the preceptor gave a medical trainee.**

Strongly disagree / disagree / slightly disagree / slightly agree / agree / strongly agree/ N/A

1 2 3 4 5 6

**How would you prefer to give a medical trainee feedback (indicate your preference):**

During the session, in-person

Immediately after the session, in a questionnaire

Either is fine

**If you gave feedback on a medical trainee, who do you think should see it?**

Only the student / Only the teacher / Both student and teacher

Please select what you think are the 5 most important options.

If I were giving feedback to a medical student or resident, I would want to comment on if the trainee:

Is skillful at examining

 Explains things clearly

 Involves me in the decisions about the medical plans

 Is confident

 Did or said anything that made me uncomfortable

 Is likeable

 Is organized and efficient during the appointment

 Is respectful

 Makes me feel at ease

 Addresses my concerns and takes them seriously

 Listens and gives me their full attention

 Is knowledgeable

**Which other qualities of a doctor are important to you** ?_________________

**Tell us a little bit about yourself:**

What is your age? _____ years

What is your gender? ______male ______female _____other (please specify) ______prefer not to say

Which ethnic background do you identify with: ______________________

Where do you live? _____city (urban) ______rural ______other (please specify) _____

What is your highest level of education?

Less than Grade 12

Grade 12

GED

Trades or Business Certificate

Bachelor

Masters

PhD

Other (please specify)

**Medical Learner Survey**

**PART A**

**I am a:**

Medical Student Pediatric Resident Family Medicine Resident

In which year? ________

**What is your gender? ______male ________female _______Other (please specify) ______prefer not to say**

**Have you ever encountered a pediatric patient (ages 0-18) in clinic, or during a clinical learning session?**

**YES NO**

If yes, was the patient’s caregivers present?

Yes

No

**During any clinical encounter with real patients (not simulated) of any age, approximately how many times has a preceptor specifically asked the patient how you did or how you could improve?**

Yes

No

If yes, about how often do you recall?

Rarely

Sometimes

Often

Always

**How much do you agree with the following statements:**

**Receiving patient feedback would allow me to improve my patient-centered clinical skills (ex. communication, empathy, etc., NOT technical skills).**

Strongly disagree / disagree / slightly disagree / slightly agree / agree / strongly agree/ N/A

1 2 3 4 5 6

**If a patient gives feedback on my performance in a questionnaire, I would want to see it.**

Strongly disagree / disagree / slightly disagree / slightly agree / agree / strongly agree/ N/A

1 2 3 4 5 6

**If a patient gives feedback, ONLY the student should see the feedback, NOT the preceptor or College of Medicine.**

Strongly disagree / disagree / slightly disagree / slightly agree / agree / strongly agree/ N/A

1 2 3 4 5 6

**Knowing I could get the patient’s feedback would probably improve how I interact with the next patient.**

Strongly disagree / disagree / slightly disagree / slightly agree / agree / strongly agree/ N/A

1 2 3 4 5 6

**Patient feedback on my clinical performance is unique and provides valuable and unique information for skill development beyond what the preceptor can provide.**

Strongly disagree / disagree / slightly disagree / slightly agree / agree / strongly agree/ N/A

1 2 3 4 5 6

**I believe patient feedback could make me a better doctor in the future.**

Strongly disagree / disagree / slightly disagree / slightly agree / agree / strongly agree/ N/A

1 2 3 4 5 6

**Positive patient feedback would be a valuable addition to the Medical Student Performance Letter (MSPR)/Dean’s letter.**

Strongly disagree / disagree / slightly disagree / slightly agree / agree / strongly agree/ N/A

1 2 3 4 5 6

**If patient feedback was included in my evaluation, this will provide a more comprehensive picture of my clinical skills.**

Strongly disagree / disagree / slightly disagree / slightly agree / agree / strongly agree/N/A

1 2 3 4 5 6

**For my classmates who struggles with positive patient interactions, patient feedback would help them improve.**

Strongly disagree / disagree / slightly disagree / slightly agree / agree / strongly agree/ N/A

1 2 3 4 5 6

**If I were a patient, I too would like the chance to give (anonymous) feedback to trainees who have seen me.**

Strongly disagree / disagree / slightly disagree / slightly agree / agree / strongly agree/ N/A

1 2 3 4 5 6

**I feel that ONLY preceptors, NOT patients can evaluate my clinical performance, even in areas outside of technical skill (ex. communication, empathy, etc).**

Strongly disagree / disagree / slightly disagree / slightly agree / agree / strongly agree/ N/A

1 2 3 4 5 6

**Knowing I may get feedback from the patient would probably worsen my performance in clinic.**

Strongly disagree / disagree / slightly disagree / slightly agree / agree / strongly agree/ N/A

1 2 3 4 5 6

**Regular patient feedback should also be given to physicians in practise, not just trainees.**

Strongly disagree / disagree / slightly disagree / slightly agree / agree / strongly agree/ N/A

1 2 3 4 5 6

**I think patients would be honest when giving feedback on my clinical skills, if they were using an anonymous questionnaire after the session.**

Strongly disagree / disagree / slightly disagree / slightly agree / agree / strongly agree/ N/A

1 2 3 4 5 6

**I would be MORE likely to change my communication skills if I got feedback from a patient, than if the feedback came from a doctor.**

Strongly disagree / disagree / slightly disagree / slightly agree / agree / strongly agree/ N/A

1 2 3 4 5 6

**A numerical score alone is not very helpful in measuring my skills. I would also want some narrative context to the score.**

Strongly disagree / disagree / slightly disagree / slightly agree / agree / strongly agree/ N/A

1 2 3 4 5 6

**I think that feedback provided by patients should be reviewed and edited to be more constructive, if necessary, before being shared with students.**

Strongly disagree / disagree / slightly disagree / slightly agree / agree / strongly agree/ N/A

1 2 3 4 5 6

**Any comments you would like to add: ______________________________________________**

Please select what you think are the 5 most important options.

What questions would you really want patients/their parents to answer regarding your performance in clinic?

Did I spend some time talking or playing with your child to get to know them better?

Did I seem skillful during the physical exam?

Did I explain things clearly?

Did I involve you in the decisions about the medical plans?

Was I confident?

Did I do or say anything that made you uncomfortable?

Was I likeable?

Was I organized and efficient during the appointment?

Was I respectful?

Did I make you feel at ease?

Did I address your concerns and take them seriously?

Did I listen and give you my full attention?

Did I seem knowledgeable?

**What else would you like to know from patients about your clinical skills**? ________________
