# Supplementary material for: Ask the Parent: Developing a Pediatric Feedback Form for Medical Learners
Source: J Med Educ Curric Dev. 2025 Mar 13;12:23821205251327375. doi: 10.1177/23821205251327375 (PMC11907601; doi:10.1177/23821205251327375)
Supplement: sj-docx-3-mde-10.1177_23821205251327375 - Supplemental material for Ask the Parent: Developing a Pediatric Feedback Form for Medical Learners [file sj-docx-3-mde-10.1177_23821205251327375.docx]

Additional File 2 2 – Additional Tables

**Ask the Parent: Developing a Client Feedback Form to Improve Medical Learners’ Pediatric Clinical Skills**

Sarah-Marie Durr^1^, Sanjida Newaz^2^, Susan Petryk^1^

*^1^Department of Medicine, University of Saskatchewan, Saskatoon, Saskatchewan, Canada.*

*^2^Research Department, Saskatchewan Health Authority, Saskatchewan, Canada*

Table 1: Parent’s agreement or disagreement on providing feedback to medical learners.

|  | Strongly Disagree | Disagree | Slightly Disagree | Slightly Agree | Agree | Strongly Agree | N/A | Missing data |
| --- | --- | --- | --- | --- | --- | --- | --- | --- |
| **Patients and/or their parents should give medical trainees feedback about what they liked and what they could do better to help them become better doctors. Feedback should NOT only come from other doctors or teachers.** | 4 (4.6%) | 0 | 2 (2.3%) | 8 (9.2%) | 28 (32.2%) | 36 (41.4%) | 2 (2.3%) | 7 |
| **I think ONLY doctors should give feedback to medical trainees, and NOT patients or their parents.** | 30 (34.5%) | 19 (21.8%) | 15 (17.2%) | 10 (11.5%) | 3 (3.4%) | 3 (3.4%) | 0 | 7 |
| **I have seen some medical trainees who could really use my feedback to improve their interaction skills.** | 9 (10.3%) | 13 (14.9%) | 7 (8%) | 15 (17.2%) | 18 (20.7%) | 8 (9.2%) | 11 (12.6%) | 6 |
| **There have been times when I wanted to give feedback to a medical trainee but didn't because I was not asked.** | 11 (12.6%) | 18 (20.7%) | 10 (11.5%) | 12 (13.8%) | 14 (16.1%) | 2 (2.3%) | 13 (14.9%) | 7 |
| **I would be willing to fill out a 2-minute anonymous questionnaire on a medical trainee's performance right after an appointment.** | 4 (4.6%) | 4 (4.6%) | 2 (2.3%) | 9 (10.3%) | 30 (34.5%) | 28 (32.2%) | 4 | 6 |
| **I might not be fully honest when giving direct, in-person feedback to the medical trainee because I do not want to hurt their feelings.** | 14 (16.1%) | 15 (17.2%) | 7 (8%) | 17 (19.5%) | 16 (18.4%) | 10 (11.5%) | 2 (2.3%) | 6 |
| **I would be completely honest in my feedback, even if it was negative, if I knew it would be anonymous.** | 2 (2.3%) | 3 (3.4%) | 4 (4.6%) | 9 (10.3%) | 23 (26.4%) | 38 (43.7%) | 2 (2.3%) | 6 |
| **If my child was very sick, I would NOT want to fill out a 2-minute questionnaire on a medical trainee's performance.** | 9 (10.3%) | 9 (10.3%) | 7 (8%) | 12 (13.8%) | 27 (31%) | 15 (17.2%) | 2 (2.3%) | 6 |

Table 2: SP’s agreement or disagreement on providing feedback to medical learners.

|  | Strongly disagree | Disagree | Slightly disagree | Slightly agree | Agree | Strongly agree | N/A |
| --- | --- | --- | --- | --- | --- | --- | --- |
| **Simulates patients should give feedback** | 1 (0.8%) | 1 (0.8%) | 2 (1.5%) | 24 (18%) | 41 (30.8%) | 64 (48.1%) | 0 |
| **I think ONLY doctors should give feedback** | 46 (34.6%) | 47 (35.3%) | 26 (19.5%) | 7 (5.3%) | 5 (3.8%) | 1 (0.8%) | 1 |
| **I have seen some medical trainees who could really use my feedback** | 4 (3.0%) | 1 (0.8%) | 4 (3.0%) | 35 (26.3%) | 47 (35.3%) | 34 (25.6%) | 7 |
| **There have been times when I wanted to give feedback to a medical trainee but didn't because I was not asked** | 6 (4.5%) | 12 (9.0%) | 6 (4.5%) | 23 (17.3%) | 45 (33.8%) | 31 (23.3%) | 10 |
| **I would NOT want to give my honest feedback to the medical trainee if my opinion was different from what their preceptor said** | 18 (13.5%) | 47 (35.3%) | 22 (16.5%) | 23 (17.3%) | 12 (9.0%) | 5 (3.8%) | 4 |
| **I would be willing to fill out a 2-minute questionnaire on a medical trainee's performance right after a session.** | 1 (0.8%) | 2 (1.5%) | 3 (2.3%) | 17 (12.8%) | 56 (42.1%) | 50 (37.6%) | 4 |
| **I would be comfortable disagreeing with the feedback that the preceptor gave a medical trainee** | 12 (9.0%) | 13 (9.8%) | 19 (14.3%) | 32 (24.1%) | 37 (27.8%) | 13 (9.8%) | 7 |

Table 3: Medical learners’ agreement or disagreement on receiving feedback from patients and/or parents

|  | Strongly disagree | Disagree | Slightly disagree | Slightly agree | Agree | Strongly agree | N/A |
| --- | --- | --- | --- | --- | --- | --- | --- |
| **Receiving patient feedback would allow me to improve my patient-centered clinical skills (ex. communication, empathy, etc., NOT technical skills).** | 1 | 0 | 1 | 6 | 29 | 25 | 1 |
| **If a patient gives feedback on my performance in a questionnaire, I would want to see it** | 2 | 0 | 0 | 2 | 25 | 33 | 1 |
| **If a patient gives feedback, ONLY the student should see the feedback, NOT the preceptor or the College of Medicine.** | 4 | 11 | 11 | 17 | 5 | 14 | 1 |
| **Knowing I could get the patient's feedback would probably improve how I interact with the next patient.** | 3 | 5 | 2 | 12 | 24 | 15 | 2 |
| **Patient feedback on my clinical performance provides valuable and unique information for skill development beyond what the preceptor can provide.** | 1 | 0 | 1 | 10 | 22 | 29 | 0 |
| **I believe patient feedback could make me a better doctor in the future** | 1 | 0 | 0 | 6 | 23 | 33 | 0 |
| **Positive patient feedback would be a valuable addition to the Medical Student Performance Letter (MSPR)/Dean's letter** | 2 | 7 | 3 | 9 | 15 | 23 | 4 |
| **If patient feedback was included in my evaluation, this would provide a more comprehensive picture of my clinical skills** | 2 | 3 | 1 | 14 | 26 | 17 | 0 |
| **For my classmates who struggle with positive patient interactions, patient feedback would help them improve** | 1 | 1 | 2 | 13 | 25 | 20 | 1 |
| **If I were a patient, I too would like the chance to give (anonymous) feedback to trainees who have seen me** | 1 | 0 | 0 | 13 | 21 | 28 | 0 |
| **I feel that ONLY preceptors, NOT patients can evaluate my clinical performance, even in areas outside of technical skill (ex. communication, empathy, etc).** | 19 | 31 | 6 | 4 | 2 | 1 | 0 |
| **Knowing I may get feedback from the patient would probably worsen my performance in clinic** | 10 | 30 | 16 | 4 | 2 | 1 | 0 |
| **Regular patient feedback should also be given to physicians in practise, not just trainees** | 1 | 0 | 1 | 15 | 24 | 22 | 0 |
| **I think patients would be honest when giving feedback on my clinical skills, if they were using an anonymous questionnaire after the session** | 2 | 0 | 7 | 9 | 30 | 15 | 0 |
| **I would be MORE likely to change my communication skills if I got feedback from a patient, than if the feedback came from a doctor** | 3 | 12 | 21 | 16 | 6 | 4 | 1 |
| **A numerical score alone is not very helpful in measuring my skills. I would also want some narrative context to the score** | 2 | 0 | 1 | 7 | 30 | 23 | 0 |
| **I think that feedback provided by patients should be reviewed and edited to be more constructive, if necessary, before being shared with students** | 8 | 9 | 12 | 14 | 13 | 6 | 1 |
